# Endoscopic ultrasound-guided treatment of splenic artery pseudoaneurysm and pancreatic pseudocyst

**DOI:** 10.1055/a-2727-0211

**Published:** 2025-12-04

**Authors:** Fengxin Wang, Baobao Wang, Zhenjun Wang, Guan-Jun Kou, Ning Zhong

**Affiliations:** 1 91623 Department of Gastroenterology, Qilu Hospital of Shandong University, Jinan, China; 2 91623 Shandong Provincial Clinical Research Center for digestive disease, Qilu Hospital of Shandong University, Jinan, China

A 37-year-old man with a 1-year history of intermittent abdominal pain was transferred to our hospital. Contrast-enhanced computed tomography (CT) revealed features of chronic pancreatitis, including diffuse pancreatic calcifications, dilation of the pancreatic duct, and a pancreatic pseudocyst (PPC). Additionally, a splenic artery pseudoaneurysm (PsA) was identified inside the pseudocyst ( Fig. 1 ).

**Fig. 1:**
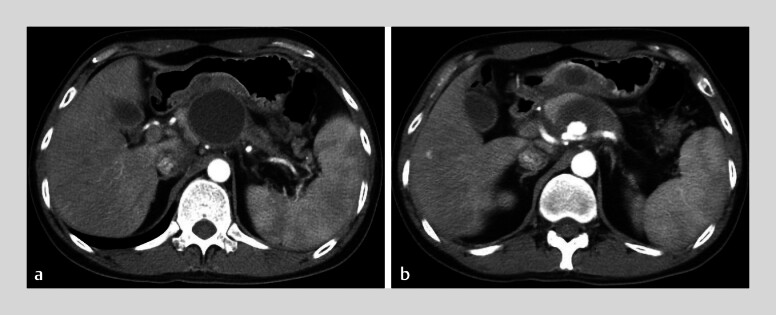
Contrast-enhanced computed tomography images showing: **a** Pancreatic pseudocyst; **b** A splenic artery pseudoaneurysm inside the pseudocyst.

Endoscopic ultrasound (EUS) revealed a well-defined PsA, measuring 1.3 cm × 1.2 cm, arising from the splenic artery and located within a PPC. Doppler imaging demonstrated active arterial flow with a characteristic “to-and-fro” waveform ( Fig. 2 ). The PPC measured approximately 5 cm in diameter and was situated posterior to the gastric fundus. We first punctured into the PPC using a 19-G biopsy needle and aspirated 50 mL of dark brown fluid ( Fig. 3 ). Subsequently, the needle was exchanged for a 22-G biopsy needle (G31521, Cook Medical, USA), through this access, a 10 mm × 10 mm Tornado embolization microcoil was deployed, followed by injection of a mixture consisting of 1 mL cyanoacrylate glue and 1 ml distilled water, achieving complete PsA occlusion ( Fig. 4 , Video 1 ). Subsequently, the 19-G biopsy needle was reinserted to place a guidewire. Then, the gastric and cyst walls were incised with a cystotome (CST-10, Cook Medical, Ireland), and a 7-Fr double-pigtail nasocystic drainage catheter (PBD-V813W-07, Olympus, Japan) was positioned for continuous drainage.

**Fig. 2:**
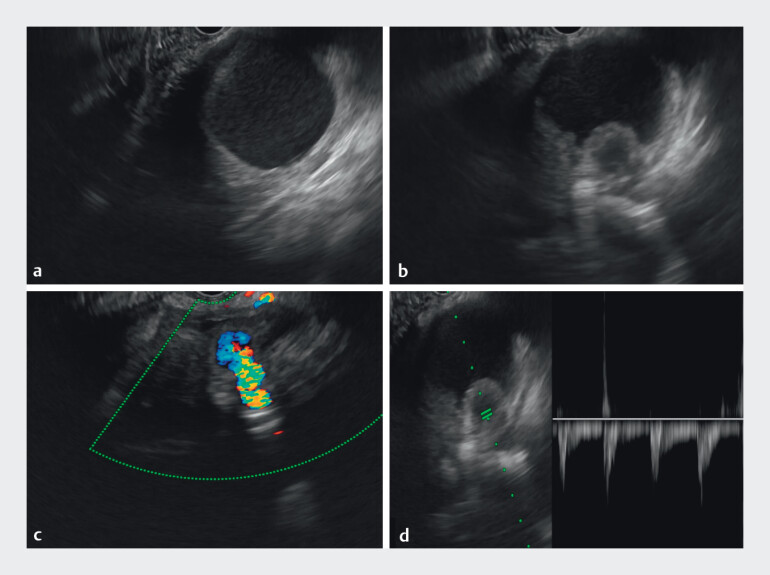
The PPC measured approximately 5 cm in diameter. Endoscopic ultrasound images showing a 1.3 cm × 1.2 cm splenic artery pseudoaneurysm from the splenic artery with a characteristic “to-and-fro” waveform on Doppler.

**Fig. 3:**
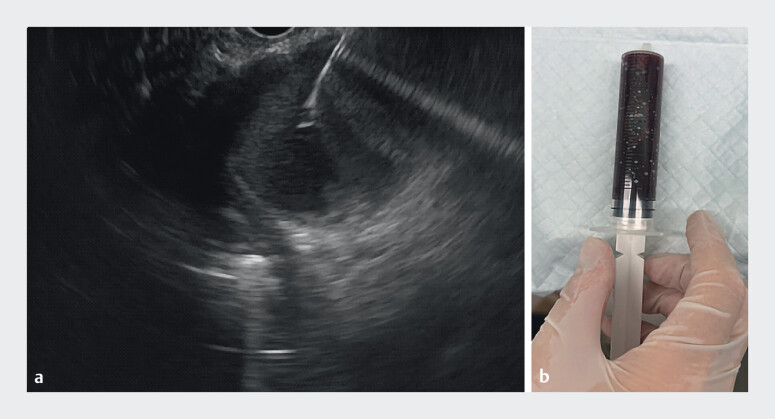
**a** Puncture into the pancreatic pseudocyst using a 19-G biopsy needle; **b** Aspirate 50 mL of dark brown fluid.

**Fig. 4:**
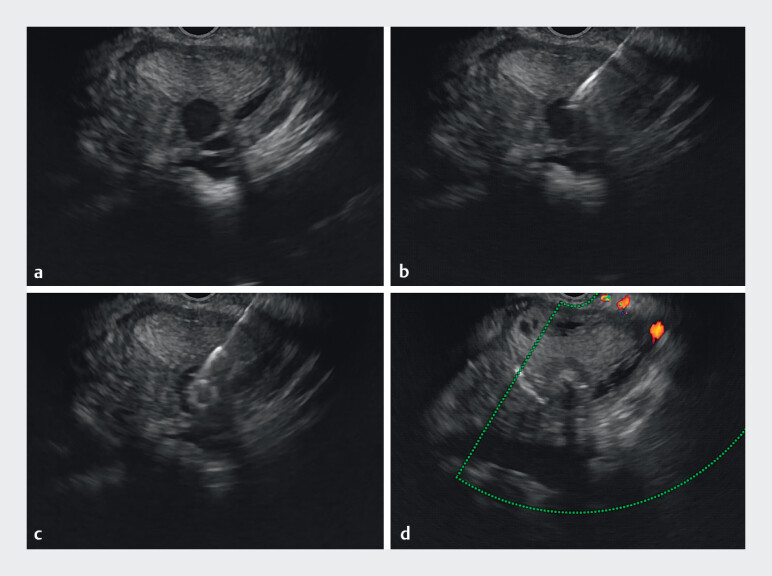
EUS-guided treatment of a splenic artery pseudoaneurysm: **a** A splenic artery pseudoaneurysm; **b** Replace with a 22-G biopsy needle; **c** Injection of cyanoacrylate glue into the pseudoaneurysm; **d** Complete embolization of the pseudoaneurysm.

EUS-guided treatment of splenic artery pseudoaneurysm and pancreatic pseudocyst.Video 1

The X-ray shows the drainage catheter is in the normal position. During postoperative follow-up, CT imaging demonstrated proper positioning of the coils within the PsA, without evidence of splenic infarction ( Fig. 5 ).

**Fig. 5:**
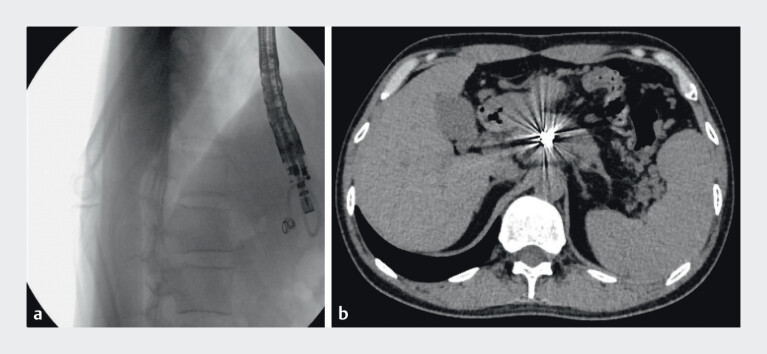
**a** Double-pigtail nasocystic drainage catheter in the correct position; **b** Computed tomography imaging demonstrated proper positioning of the coils within the PsA, without evidence of splenic infarction.

PPC and PsA are common complications of pancreatitis 1 . PsA is associated with a rupture risk of up to 40%. Upon rupture, it may lead to life-threatening hemorrhage, with mortality rates reaching as high as 90% 2 . This case provides a more effective and efficient treatment option for patients with concomitant PPC and PsA.

Endoscopy_UCTN_Code_TTT_1AS_2AD

## Notes

Endoscopy E-Videos https://eref.thieme.de/e-videos
*E-Videos* is an open access online section of the journal *Endoscopy* , reporting on interesting cases and new techniques in gastroenterological endoscopy. All papers include a high-quality video and are published with a Creative Commons CC-BY license. Endoscopy E-Videos qualify for HINARI discounts and waivers and eligibility is automatically checked during the submission process. We grant 100% waivers to articles whose corresponding authors are based in Group A countries and 50% waivers to those who are based in Group B countries as classified by Research4Life (see: https://www.research4life.org/access/eligibility/ ). This section has its own submission website at https://mc.manuscriptcentral.com/e-videos .
